# Supplementary material for: Longitudinal gut microbiota composition of South African and Nigerian infants in relation to tetanus vaccine responses
Source: Microbiol Spectr. 2024 Jan 17;12(2):e03190-23. doi: 10.1128/spectrum.03190-23 (PMC10846250; doi:10.1128/spectrum.03190-23)
Supplement: Fig. S1 — α-diversity of meconium samples differs significantly by study site. [file spectrum.03190-23-s0001.pdf]

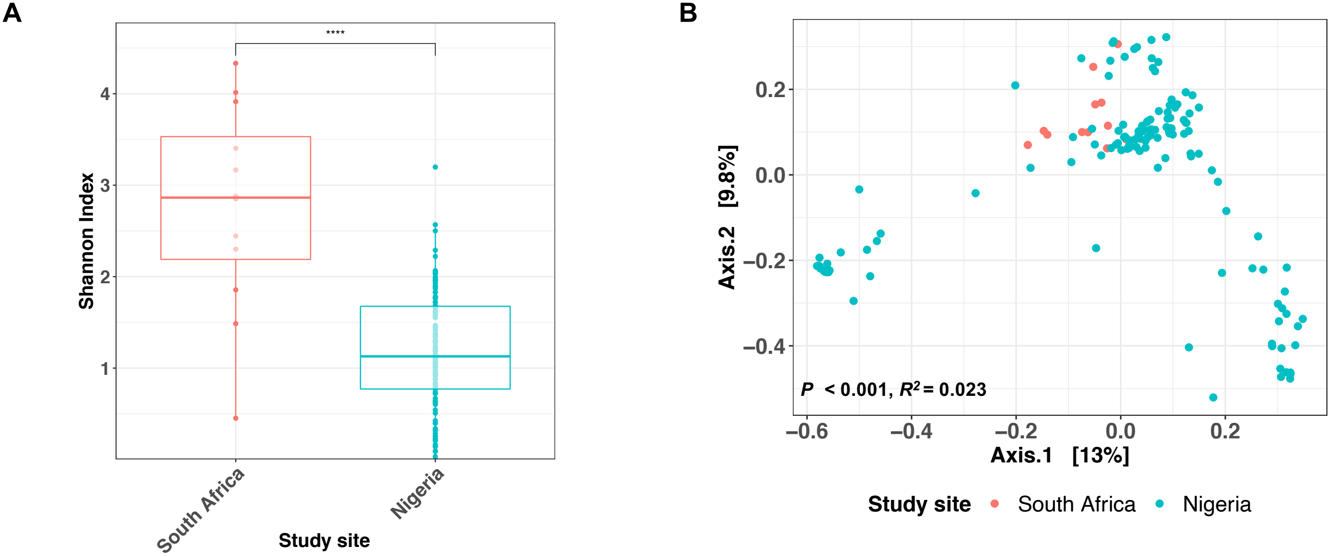

Supplementary Figure 1: α-diversity of meconium samples differs significantly by study site.
