# Supplementary material for: Longitudinal gut microbiota composition of South African and Nigerian infants in relation to tetanus vaccine responses
Source: Microbiol Spectr. 2024 Jan 17;12(2):e03190-23. doi: 10.1128/spectrum.03190-23 (PMC10846250; doi:10.1128/spectrum.03190-23)
Supplement: Fig. S6 — Association of infant anti-tetanus titer with age, HIV exposure status and mother's anti-tetanus titer. [file spectrum.03190-23-s0006.pdf]

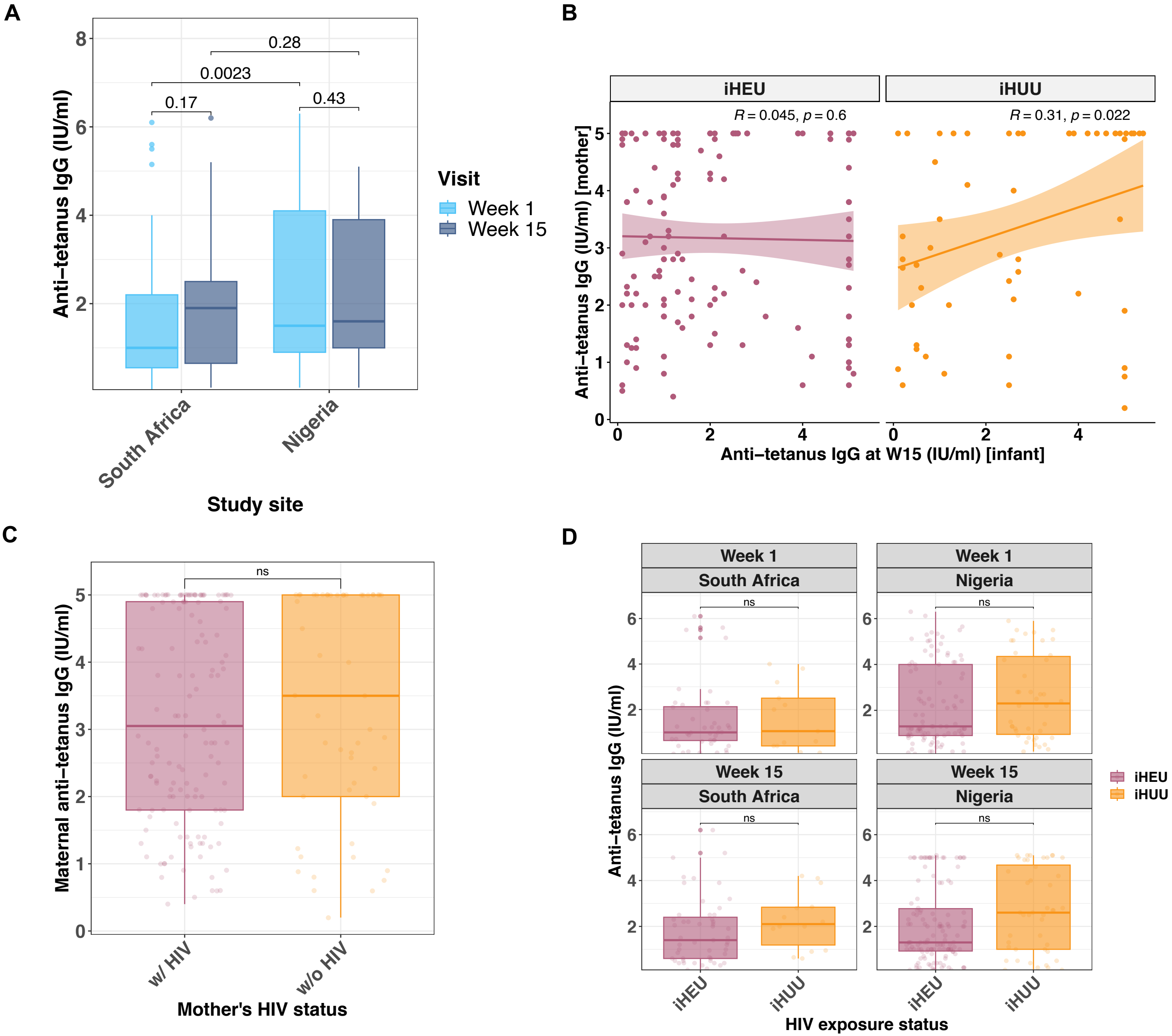

Supplementary Figure 6: Association of infant anti-tetanus titre with age, HIV exposure status and mother's anti-tetanus titre.
