# Supplementary figures and images for: Longitudinal gut microbiota composition of South African and Nigerian infants in relation to tetanus vaccine responses
Source: Microbiol Spectr. 2024 Jan 17;12(2):e03190-23. doi: 10.1128/spectrum.03190-23 (PMC10846250; doi:10.1128/spectrum.03190-23)

A

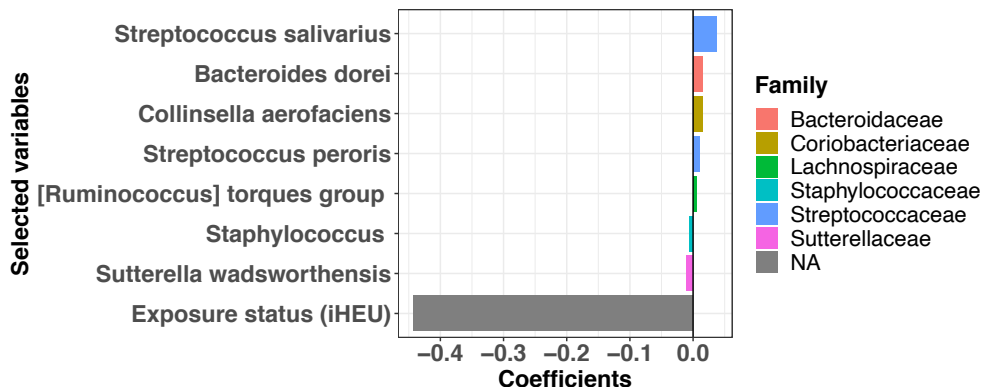

B

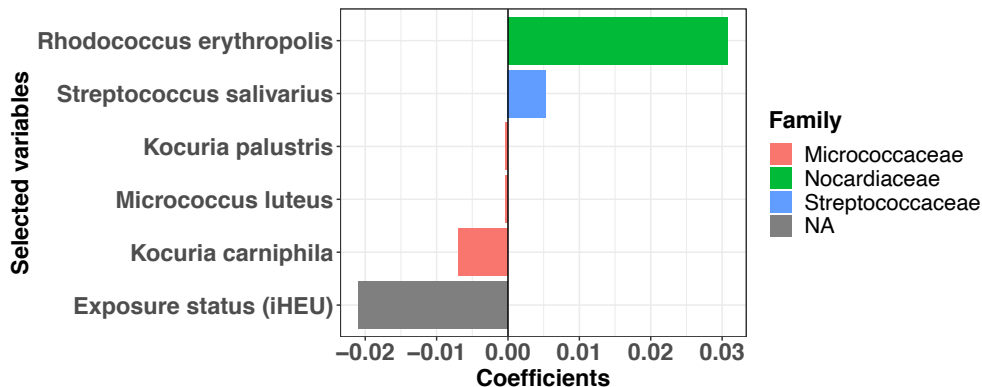

Supplement: Fig. S7 — Maternal antibodies may mask the effect of HIV exposure and microbiota on infant vaccine response. [file spectrum.03190-23-s0007.pdf]
