# Supplementary material for: Longitudinal gut microbiota composition of South African and Nigerian infants in relation to tetanus vaccine responses
Source: Microbiol Spectr. 2024 Jan 17;12(2):e03190-23. doi: 10.1128/spectrum.03190-23 (PMC10846250; doi:10.1128/spectrum.03190-23)
Supplement: SupplementalMaterials — Supplemental tables and figure legends. [file spectrum.03190-23-s0008.docx]

**Supplemental information**

**Supplementary Figure S1**

**α-diversity of meconium samples differs significantly by study site.** (A) Comparison of α-diversity (Shannon index) of meconium samples collected at day one of life between South African (n = 12) and Nigerian infants (n = 135). (B) PCoA and PERMANOVA (Bray-Curtis dissimilarity) of gut microbiota of meconium samples at day one of life (n = 147), coloured by study site. PCoA, principal coordinate analysis; PERMANOVA, permutational multivariate analysis of variance. **** *P* < 0.0001.

**Supplementary Figure S2**

**α- and β-diversity significantly differ between the countries in exclusively breastfed infants.** (A) Comparison of α-diversity (Shannon index) between EBF South African (n = 40) and Nigerian (n = 172) infants at 15 weeks of age. (B) PCoA and PERMANOVA (Bray-Curtis dissimilarity) of gut microbiota among EBF infants at 15 weeks of age (n = 212), coloured by study site. EBF, exclusively breastfed; PCoA, principal coordinate analysis; PERMANOVA, permutational multivariate analysis of variance. **** *P* < 0.0001.

**Supplementary Figure S3**

**α- and β-diversity significantly differ between the countries in vaginally delivered infants.** (A) Comparison of α-diversity (Shannon index) between vaginally delivered South African (n = 82) and Nigerian (n = 167) infants at week 1 and week 15. (B) PCoA and PERMANOVA (Bray-Curtis dissimilarity) of gut microbiota among vaginally delivered infants (n = 249) at 1 week and 15 weeks of age, coloured by study site. PCoA, principal coordinate analysis; PERMANOVA, permutational multivariate analysis of variance. **** *P* < 0.0001.

**Supplementary Figure S4**

**HIV exposure has a subtle effect on gut microbiota across two African countries.** (A) Comparison of α-diversity (Shannon index) between iHEU (n = 202) and iHUU (n = 76) at each time point by study sites. (B) PCoA and PERMANOVA (Bray-Curtis dissimilarity) of gut microbiota at 1 week and 15 weeks of age, coloured by HIV exposure status (iHEU, n = 202; iHUU, n = 76) and shaped by the study site. (C) Heatmap of the top 25 taxa in the gut microbiota of South African (n = 82) and Nigerian (n = 196) infants at 1 week and 15 weeks of age. Study site, study visit, HIV exposure status, and community cluster types (based on PAM clustering; *k* = 3) are annotated. Bacterial taxa are annotated at the species level. Different ASV IDs with identical bacterial taxa are merged. (D) Alluvial plot showing the transition of cluster groups from week 1 to week 15 at each study site (South African, n = 82; Nigerian, n = 196). Samples are grouped by PAM clustering (*k* = 3). HIV exposure status is indicated by colour. iHEU, infants who are HIV-exposed uninfected; iHUU, infants who are HIV-unexposed uninfected; PCoA, principal coordinate analysis; PERMANOVA, permutational multivariate analysis of variance; PAM, partitioning around medoids; ASVs, amplicon sequence variants. ns, not significant.

**Supplementary Figure S5**

**The effect of α- and β-diversity of co-trimoxazole on gut microbiota is marginal.** (A) Comparison of α-diversity (Shannon index) based on record of reported co-trimoxazole treatment among South African iHEU at 15 weeks of age (n = 51) (B) PCoA and PERMANOVA (Bray-Curtis dissimilarity) of gut microbiota among South African iHEU at 15 weeks of age (n = 51), coloured reported co-trimoxazole treatment history. PCoA, principal coordinate analysis; PERMANOVA, permutational multivariate analysis of variance.

**Supplementary Figure S6**

**Association of infant anti-tetanus titre with age, HIV exposure status and mother’s anti-tetanus titre.** (A) Comparison of infant anti-tetanus IgG titres (IU/ml) between week 1 and week 15 in South Africa (n = 77) and Nigeria (n = 192). (B) Scatter plots and Spearman’s rank correlation coefficients (R) of anti-tetanus IgG titres between Nigerian mothers (y-axis; n = 191) and their infants at week 15 (x-axis; n = 191). (C) Box plot comparing maternal anti-tetanus IgG titres by HIV status (HIV positive, n = 138; HIV negative, n = 53). (D) Comparison of anti-tetanus IgG titres between iHEU (n = 197) and iHUU (n = 72) at each time point by study sites. *P*-values comparing anti-tetanus IgG titres were adjusted for multiple comparisons using the Benjamini-Hochberg method. iHEU, infants who are HIV-exposed uninfected; iHUU, infants who are HIV-unexposed uninfected; ns, not significant; w/, with; w/o, without.

**Supplementary Figure S7**

**Maternal antibodies may mask the effect of HIV exposure and microbiota on infant vaccine response.** Rank-transformed top 50 ASVs (at either week 1 or week 15) and HIV exposure status were used as explanatory variables for the LASSO regression to assess the association with TT vaccine response at 15 weeks of age. To explore the masking effect of passively transferred maternal antibodies on the regression model, anti-tetanus IgG at week 1 data was not included in the model. Each model was constructed separately based on geographical location and time point. The optimal coefficient tuning parameter (lambda.min) was chosen using 10-fold cross-validation. Selected variables and their glmnet coefficients were plotted. Colour of the bars represents taxonomy at the family level. Week 1 ASVs and HIV exposure were associated with week 15 TT vaccine response among South African infants (A), and week 15 ASVs and HIV exposure were associated with the vaccine response among Nigerian infants (B). iHEU, infants who are HIV-exposed uninfected; ASVs, amplicon sequence variants; TT, tetanus toxoid.

**Supplementary Table S2 ANCOM-BC analysis of iHEU and iHUU living in South Africa at 15 weeks of age, adjusted for mode of feeding and reported antibiotic history**

Differentially abundant ASVs (adj *P* < 0.05) among iHEU relative to iHUU at the first week or 15 weeks of age in South Africa (n = 82). Data at week 15 were adjusted by mode of feeding and reported antibiotics history. Positive LFC values indicate higher abundance among iHEU, whereas negative LFC values indicate higher abundance among iHUU. No differentially abundant bacterial taxa were identified among Nigerian infants.

**Supplementary Table S3 Prediction of factors associated with TT vaccine response by LASSO regression**

1. The top 50 rank-transformed bacterial taxa at week 1, HIV exposure status, and anti-tetanus IgG titres at week 1 were included as explanatory variables. Coefficients of each variable after penalization with a value of lambda that gives the minimum mean of cross-validated error (lambda.min) are indicated.
2. The top 50 rank-transformed bacterial taxa at week 15 and HIV exposure status were included as explanatory variables. Coefficients of each variable after penalization with a value of lambda that gives the minimum mean of cross-validated error (lambda.min) are indicated.

**Supplementary Table S1 Comparison of characteristics by HIV exposure status**

|  |  | **iHUU** | **iHEU** | ***P*** |
| --- | --- | --- | --- | --- |
|  |  | **(N = 76)** | **(N = 202)** |  |
| **Maternal characteristics** |  |  |  |  |
| **Study site (n; %)** | South Africa | 21 (27.6) | 61 (30.2) | 0.787 |
|  | Nigeria | 55 (72.4) | 141 (69.8) |  |
| **Mother’s age at delivery (years; mean (SD))** |  | 29 (5.00) | 30 (5.57) | 0.098 |
| **Education (n; %)** | None | 0 (0.0) | 2 (1.0) | 0.002 |
|  | Elementary | 11 (14.5) | 59 (29.2) |  |
|  | Secondary | 38 (50.0) | 107 (53.0) |  |
|  | Higher | 27 (35.5) | 34 (16.8) |  |
| **Unemployed (n; %)** |  | 16 (21.1) | 49 (24.3) | 0.911 |
| **Formal housing (n; %)** |  | 61 (80.3) | 157 (77.7) | 0.548 |
| **Electricity (n; %)** |  | 70 (92.1) | 186 (92.1) | 1 |
| **Refrigerator (n; %)** |  | 48 (63.2) | 118 (58.4) | 0.561 |
| **Running water (n; %)** |  | 28 ( 36.8) | 58 (28.7) | 0.245 |
| **Marital status (n; %)** | Married/ living together | 63 (82.9) | 148 (73.3) | 0.130 |
|  | Single | 13 (17.1) | 54 (26.7) |  |
| **Gravidity (n; median [IQR])** |  | 2 [1, 3] | 2 [1, 3] | 0.071 |
| **Mother’s weight at enrollment (kg; mean (SD))^a^** |  | 66.82 (13.56) | 65.45 (12.86) | 0.443 |
| **Mother on ART at delivery (n; %)** |  | - | 199 (98.5) | - |
| **CD4 count > 250 cells/mm^3^ (n; %)^b^** |  | - | 158 (81.0) | - |
| **Viral load below detection limit (n; %)^c^** |  | - | 93 (58.1) | - |
| **Infant characteristics** |  |  |  |  |
| **Male (n; %)** |  | 38 (50.0) | 97 (48.0) | 0.873 |
| **Gestational age at delivery (weeks; median [IQR])** |  | 40.00  [39.00, 40.70] | 39.60  [38.40, 40.40] | 0.086 |
| **Vaginal delivery (n; %)** |  | 67 (88.2) | 182 (90.1) | 0.801 |
| **Wflz at W15 (median [IQR])^d^** |  | 0.70  [-0.20, 1.47] | 0.61  [-0.52, 1.50] | 0.302 |
| **Mode of feeding at W15 (n; %)** | Exclusive breastfeeding | 62 (81.6) | 182 (90.1) | 0.084 |
|  | Mixed feeding | 14 (18.4) | 20 (9.9) |  |
| **Reported antibiotics use (n; %)** | Co-trimoxazole | 0 (0.0) | 175 (86.6) | <0.001 |
|  | Other | 5 (6.6) | 0 (0.0) |  |

IQR, interquartile range; SD, standard deviation; iHEU, infants who are HIV-exposed uninfected; iHUU, infants who are HIV-unexposed uninfected; ART, antiretroviral treatment; W15, 15 weeks of age; Wflz, weight-for-length z score.

^a^Missing data from 5 participants (iHUU, n = 2; iHEU, n =3); ^b^Missing data from 7 iHEU; ^c^Missing data from 42 iHEU; ^d^Missing data from 41 participants (iHUU, n = 18; iHEU, n = 23).

**Supplementary Table S2 ANCOM-BC analysis of iHEU and iHUU living in South Africa at 15 weeks of age, adjusted for mode of feeding and reported antibiotic history**

| **Taxonomy (Genus, Species)** | **Taxon ID** | **LFC^a^** |
| --- | --- | --- |
| **At 15 weeks of age** |  |  |
| *Collinsella aerofaciens* | ASV25 | 1.15 |
| *Klebsiella quasipneumoniae* | ASV42 | 1.13 |
| *Bifidobacterium adolescentis* | ASV296 | 1.08 |
| *Streptococcus gallolyticus* | ASV44 | 1.08 |
| *Enterococcus gilvus* | ASV157 | 1.07 |
| *Lactococcus lactis* | ASV206 | 1.01 |
| *Enterococcus raffinosus* | ASV51 | 0.98 |
| *Clostridium innocuum* group (unclassified) | ASV336 | 0.93 |
| *Enterococcus raffinosus* | ASV338 | 0.89 |
| *Dorea formicigenerans* | ASV229 | 0.82 |
| *Corynebacterium propinquum* | ASV479 | 0.73 |
| *Faecalibacterium prausnitzii* | ASV103 | 0.61 |
| *Leuconostoc lactis* | ASV324 | 0.60 |
| *Granulicatella* (unclassified) | ASV681 | 0.56 |
| *Rothia mucilaginosa* | ASV264 | 0.52 |
| *Streptococcus salivarius* | ASV104 | 0.50 |
| *Streptococcus peroris* | ASV250 | 0.50 |
| *Lactobacillus vaginalis* | ASV170 | -0.51 |
| *Lactobacillus ferme*ntum | ASV141 | -0.52 |
| *Parabacteroides distasonis* | ASV216 | -0.52 |
| *Lactobacillus rhamnosus* | ASV191 | -0.55 |
| *Veillonella* (unclassified) | ASV258 | -0.55 |
| *Enterococcus gilvus* | ASV472 | -0.65 |
| *Megasphaera elsdeni*i | ASV167 | -0.70 |
| *Olsenella* (unclassified) | ASV97 | -0.73 |
| *Senegalimassilia* (unclassified) | ASV171 | -0.75 |
| *Klebsiella michiganensis* | ASV266 | -0.81 |
| *Bacteroides caccae* | ASV552 | -0.82 |
| *Prevotella* (unclassified) | ASV176 | -0.93 |
| *Veillonella parvula* | ASV503 | -0.95 |
| *Olsenella* (unclassified) | ASV120 | -2.07 |
| *Ruminococcus torques group* (unclassified) | ASV75 | -2.37 |

Differentially abundant ASVs (adj *P* < 0.05) among iHEU relative to iHUU at the first week or 15 weeks of age in South Africa (n = 82). Data at week 15 were adjusted by mode of feeding and reported antibiotics history. Positive LFC values indicate higher abundance among iHEU, whereas negative LFC values indicate higher abundance among iHUU. No differentially abundant bacterial taxa were identified among Nigerian infants.

^a^Abundance in iHEU in relation to iHUU. ANCOM-BC: Analysis of Compositions of Microbiomes with Bias Correction; LFC, log_e_ fold change; ASV, amplicon sequence variants; iHEU, infants who are HIV-exposed uninfected; iHUU, infants who are HIV-unexposed uninfected.

**Supplementary Table S3 Prediction of factors associated with TT vaccine response by LASSO regression**

**A.**The top 50 rank-transformed bacterial taxa at week 1 in South African infants, HIV exposure status, and anti-tetanus IgG titers at week 1 were included as explanatory variables.

| **Explanatory variables** | **Coefficients** |
| --- | --- |
| (Intercept) | 1.10177 |
| Anti-tetanus IgG titer at W1 | 0 |
| [W1 ASV1] *Bifidobacterium longum* | 0 |
| [W1 ASV2] *Streptococcus salivarius* | 0 |
| [W1 ASV3] *Escherichia-Shigella coli* | 0 |
| [W1 ASV4] *Bifidobacterium longum* | 0 |
| [W1 ASV5] *Enterococcus faecalis* | 0 |
| [W1 ASV6] *Collinsella aerofacien*s | 0 |
| [W1 ASV7] *Enterococcus faecium* | 0 |
| [W1 ASV8] *Bifidobacterium catenulatum* | 0 |
| [W1 ASV9] *Streptococcus lutetiensis* | 0 |
| [W1 ASV10] *Bifidobacterium breve* | 0 |
| [W1 ASV11] *Staphylococcus caprae* | 0 |
| [W1 ASV16] *Bifidobacterium bifidum* | 0 |
| [W1 ASV19] *Streptococcus* (unclassified) | 0 |
| [W1 ASV20] *Lactobacillus gasseri* | 0 |
| [W1 ASV21] *Ruminococcus gnavus* group (unclassified) | 0 |
| [W1 ASV24] *Veillonella dispar* | 0 |
| [W1 ASV25] *Collinsella aerofaciens* | 0.01536 |
| [W1 ASV26] *Escherichia-Shigella* (unclassified) | 0 |
| [W1 ASV32] *Bacteroides vulgatus* | 0 |
| [W1 ASV36] *Klebsiella quasipneumoniae* | 0 |
| [W1 ASV38] *Veillonella atypica* | 0 |
| [W1 ASV39] *Klebsiella pneumoniae* | 0 |
| [W1 ASV40] *Enterococcus* (unclassified) | 0 |
| [W1 ASV41] *Staphylococcus* (unclassified) | -0.0061 |
| [W1 ASV46] *Klebsiella variicola* | 0 |
| [W1 ASV53] *Holdemanella* (unclassified) | 0 |
| [W1 ASV55] *Bacteroides vulgatus* | 0 |
| [W1 ASV59] *Blautia obeum* | 0 |
| [W1 ASV60] *Bacteroides fragilis* | 0 |
| [W1 ASV61] *Bifidobacterium adolescentis* | 0 |
| [W1 ASV69] *Clostridium sensu stricto 1* (unclassified) | 0 |
| [W1 ASV70] *Erysipelatoclostridium ramosum* | 0 |
| [W1 ASV72] *Streptococcus peroris* | 0.01018 |
| [W1 ASV73] *Streptococcus parasanguinis* | 0 |
| [W1 ASV75] *Ruminococcus torques* group (unclassified) | 0.00485 |
| [W1 ASV76] *Sutterella wadsworthensis* | -0.01084 |
| [W1 ASV82] *Streptococcus salivarius* | 0.03789 |
| [W1 ASV83] *Bacteroides vulgatus* | 0 |
| [W1 ASV91] *Campylobacter jejuni* | 0 |
| [W1 ASV114] *Streptococcus salivarius* | 0 |
| [W1 ASV116] *Phascolarctobacterium faecium* | 0 |
| [W1 ASV155] *Arthrobacter* (unclassified) | 0 |
| [W1 ASV159] *Veillonella parvula* | 0 |
| [W1 ASV161] *Lactobacillus gasseri* | 0 |
| [W1 ASV175] *Clostridium sensu stricto 1 butyricum* | 0 |
| [W1 ASV182] *Bacteroides dorei* | 0.01568 |
| [W1 ASV194] *Clostridium sensu stricto 1* (unclassified) | 0 |
| [W1 ASV239] *Prevotella copri* | 0 |
| [W1 ASV330] *Bifidobacterium dentium* | 0 |
| [W1 ASV603] *Bordetella pseudohinzii* | 0 |
| HIV-exposure status (iHEU) | -0.44265 |

3B. The top 50 rank-transformed bacterial taxa at week 15 in Nigerian infants and HIV exposure status were included as explanatory variables.

| **Explanatory variables** | **Coefficients** |
| --- | --- |
| (Intercept) | 1.207 |
| [W15 ASV1] *Bifidobacterium longum* | 0 |
| [W15 ASV2] *Streptococcus salivarius* | 0.00532 |
| [W15 ASV3] *Escherichia-Shigella coli* | 0 |
| [W15 ASV4] *Bifidobacterium longum* | 0 |
| [W15 ASV5] *Enterococcus faecalis* | 0 |
| [W15 ASV6] *Collinsella aerofaciens* | 0 |
| [W15 ASV7] *Enterococcus faecium* | 0 |
| [W15 ASV9] *Streptococcus lutetiensis* | 0 |
| [W15 ASV10] *Bifidobacterium breve* | 0 |
| [W15 ASV11] *Staphylococcus caprae* | 0 |
| [W15 ASV12] *Staphylococcus saprophyticus* | 0 |
| [W15 ASV13] *Kocuria carniphila* | -0.00694 |
| [W15 ASV15] *Staphylococcus aureus* | 0 |
| [W15 ASV16] *Bifidobacterium bifidum* | 0 |
| [W15 ASV18] *Staphylococcus haemolyticus* | 0 |
| [W15 ASV19] *Streptococcus* (unclassified) | 0 |
| [W15 ASV22] *Staphylococcus saprophyticus* | 0 |
| [W15 ASV23] *Pediococcus pentosaceus* | 0 |
| [W15 ASV26] *Escherichia-Shigella* (unclassified) | 0 |
| [W15 ASV28] *Bifidobacterium longum* | 0 |
| [W15 ASV29] *Pseudomonas azotoformans* | 0 |
| [W15 ASV30] *Kocuria palustris* | -0.00028 |
| [W15 ASV31] *Micrococcus luteus* | -0.00037 |
| [W15 ASV35] *Libanicoccus* (unclassified) | 0 |
| [W15 ASV36] *Klebsiella quasipneumoniae* | 0 |
| [W15 ASV37] *Staphylococcus equorum* | 0 |
| [W15 ASV43] *Staphylococcus haemolyticus* | 0 |
| [W15 ASV45] *Rhodococcus erythropolis* | 0.03079 |
| [W15 ASV48] *Jeotgalicoccus* (unclassified) | 0 |
| [W15 ASV49] *Oceanobacillus oncorhynchi* | 0 |
| [W15 ASV57] *Globicatella* (unclassified) | 0 |
| [W15 ASV67] *Staphylococcus haemolyticus* | 0 |
| [W15 ASV68] *Staphylococcus haemolyticus* | 0 |
| [W15 ASV74] *Brevundimonas mediterranea* | 0 |
| [W15 ASV78] *Micrococcus luteus* | 0 |
| [W15 ASV80] *Enterococcus gallinarum* | 0 |
| [W15 ASV81] *Enterococcus faecalis* | 0 |
| [W15 ASV85] *Brachybacterium* (unclassified) | 0 |
| [W15 ASV86] *Oceanobacillus* (unclassified) | 0 |
| [W15 ASV88] *Enterococcus faecalis* | 0 |
| [W15 ASV94] *Oceanobacillus profundus* | 0 |
| [W15 ASV111] *Clostridium sensu stricto 1* (unclassified) | 0 |
| [W15 ASV129] *Macrococcus caseolyticus* | 0 |
| [W15 ASV131] *Staphylococcus lentus* | 0 |
| [W15 ASV187] *Staphylococcus* (unclassified) | 0 |
| [W15 ASV202] *Enterococcus casseliflavus* | 0 |
| [W15 ASV255] *Staphylococcus kloosii* | 0 |
| [W15 ASV294] *Dietzia* (unclassified) | 0 |
| [W15 ASV310] *Staphylococcus succinus* | 0 |
| [W15 ASV582] *Staphylococcus sciuri* | 0 |
| HIV-exposure status (iHEU) | -0.02089 |

Coefficients of each variable after penalization with a value of lambda that gives the minimum mean of cross-validated error (lambda.min) are indicated.

TT, tetanus toxoid; W15, W1, 1 week of age, 15 weeks of age; ASV, amplicon sequence variant; iHEU, infants who are HIV-exposed uninfected; LASSO, Least Absolute Shrinkage and Selection Operator.

Coefficients of each variable after penalization with a value of lambda that gives the minimum mean of cross-validated error (lambda.min) are indicated.
